# Diaphragmatic Hernia in a Preterm with Congenital Heart Defects with Successful Outcome: A Case Report

**DOI:** 10.31729/jnma.7462

**Published:** 2022-06-30

**Authors:** Sunil Raja Manandhar, Ashish Lal Shrestha, Sabina Shrestha, Rydam Basnet

**Affiliations:** 1 Neonatology Unit, Department of Pediatrics, Kathmandu Medical College and Teaching Hospital, Sinamangal, Kathmandu, Nepal; 2 Department of Pediatric and Neonatal Surgery, Kathmandu Medical College and Teaching Hospital, Sinamangal, Kathmandu, Nepal

**Keywords:** *congenital diaphragmatic hernia*, *preterm*, *surgical repair*, *ventricular septal defect*

## Abstract

Congenital diaphragmatic hernia is an anatomical defect of the diaphragm that is often associated with serious cardiopulmonary complications. It can also be associated with many other problems like multi systemic anomalies, chromosomal aneuploidy and prematurity. Of these, cardiac defects, liver herniation and prematurity seem to have a pivotal role in affecting the outcomes of repair. We hereby present a preterm newborn with such multiple defects repaired on 15^th^ day of life and post operatively managed in a Neonatal Intensive Care Unit with a successful outcome. The key learning objective for our team in this case was to identify the steps taken that led to a successful management of a low birth weight preemie with multiple defects in a resource limited set up.

## INTRODUCTION

The development of diaphragm occurring between 3-9 weeks of life involves the fusion of septum transversum, esophageal mesentery, pleuro-peritoneal membrane and lateral muscular components of the body wall.^1,2^ A congenital defect in this leads to Congenital Diaphragmatic Hernia (CDH) wherein herniated abdominal viscera cause serious cardiopulmonary complications.^1^ While majority with isolated CDH present with pulmonary hypoplasia and persistent pulmonary hypertension, multi-system affectations can be associated. Of these cardiac, gastrointestinal, and genitourinary anomalies cause significant morbidity.^3^ Prematurity, however is considered the strongest factor affecting outcomes despite a successful repair.^4^ We present a preterm with multiple defects repaired surgically with a successful outcome.

## CASE REPORT

**Figure 1:**
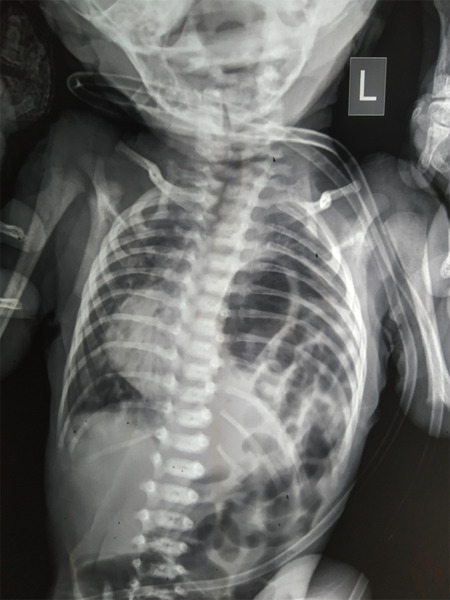
Chest radiograph showing loops of gas filled bowel over the left side of chest suggestive of left sided CDH.

A preterm male baby delivered from a singleton pregnancy at 35 weeks of gestation elsewhere with a birth weight of 1890 grams was brought in to the emergency department at 14 days of life with complaints of poor feeding and fast breathing. He was admitted to the Neonatal Intensive Care Units (NICU) for respiratory stabilization wherein the chest radiograph revealed a left sided CDH (Figure 1).

**Figure 2:**
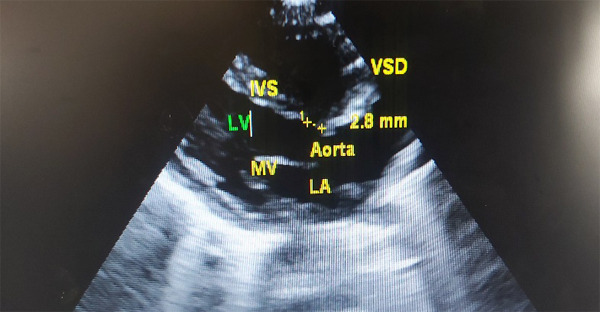
2D echocardiography showing a 2.8 mm Ventricular Septal Defect.

A bedside 2D echocardiography showed a 4 mm Atrial Septal Defect (ASD) with peri-membranous 2.8 mm Ventricular Septal Defect (VSD) (Figure 2). The pulmonary artery pressure, was however, found to be within normal limits.

**Figure 3:**
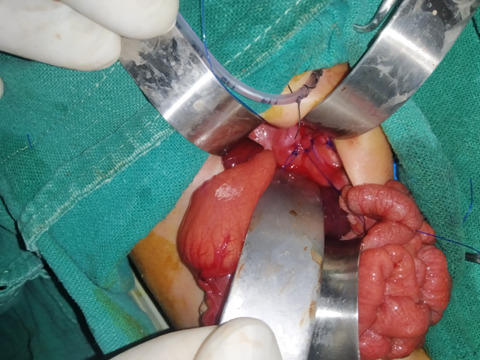
Intra-operative repair of left CDH with intercostal drain in situ.

He was subsequently intubated, maintained on mechanical ventilation and taken for laparotomy and repair of CDH the following day. At operation, a 7x3 cm postero lateral defect was found over the left hemi diaphragm with herniation of stomach, spleen, loops of jejunum, transverse colon and the left lobe of liver into the left thoracic cavity. The underlying lung however did not look grossly hypoplastic. The herniated viscera were repositioned into the abdomen and the defect was repaired with permanent sutures leaving an inter costal drain in situ (Figure 3).

**Figure 4:**
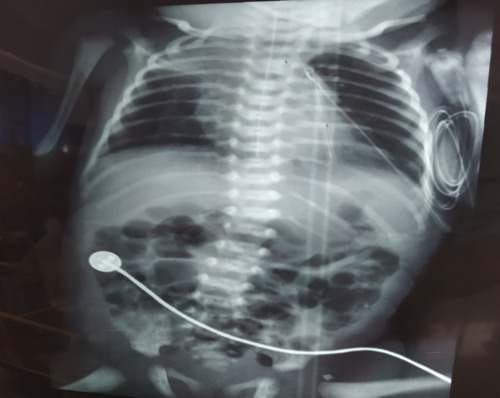
Third post-operative day chest radiograph showing adequate lung expansion and a right upper zone patch.

Postoperatively, mechanical ventilation was continued for 5 days before he could be switched over to bubble Continuous Positive Airway Pressure (CPAP) that was continued for 5 more days. The repeated chest radiograph on 3^rd^ postoperative day showed satisfactory lung expansion and a patch over the right upper lung zone (Figure 4).

With antibiotics and respiratory supports, gradual improvement in oxygenation was noted leading to tapering off of respiratory supports over the next few days. Tube feedings were initiated alongside prokinetic agents and postural management in view of anticipated gastro esophageal reflux that is a common association with CDH. Once this was tolerated, feeds were gradually upgraded to on demand breast feeds while continuing supportive measures for respiratory optimization. He was successfully discharged on 29 days of life.

Follow up visits at 2 weeks and 6 months were satisfactory with adequate weight gain and development.

## DISCUSSION

The survival of newborns with CDH depends not only on successful repair but also on several associated confounding factors such as prematurity, liver herniation and associated cardiopulmonary defects.

Prematurity that is steadily increasing over the last decade seems to be a major determinant of survival in newborns with CDH.^5^ In a series of 5,068 newborns with CDH, from an advanced center following a successful repair, the overall survival rate was found to be only 68.7%. Despite progresses in neonatal critical care this was noted to worsen further with decreasing gestational age (73.1% in term as compared to 53.5% in preterm).^6^

Similarly, liver herniation was associated with a worse prognosis with earlier studies reporting only 56% survival as opposed to near 100% without it. A metaanalysis had reported an overall decrease in survival from 74% to 45% with liver herniation.^7,8^

Likewise, the duration of mechanical ventilation and possibilities of early weaning are determined by the degree of pulmonary hypoplasia that follows an inverse relationship affecting the survival outcome.^9^

Our patient had liver herniation as a part of multi visceral involvement through the diaphragmatic defect that was successfully repaired, in addition to prematurity. He however did not have clinico-radiological or intra operative evidence of pulmonary hypoplasia and therefore could be successfully weaned after five days of ventilation.

Additionally, CDH newborns (approximately 12-25%) are at an increased risk of congenital heart defects, the most common being ASD and VSD.^10,11^ Despite advances in prenatal imaging technology, the greater availability of Pediatric Cardiac Surgery and Extra-Corporeal Membrane Oxygenation (ECMO), the prognosis of CDH with heart defects continues to remain poor.^12,13^ In our patient, a 4 mm ASD and 2.8 mm VSD were noted but he continued to remain hemodynamically stable and responded well to supportive measures.

In summary, CDH is a congenital condition that can have multiple associated anomalies which need timely recognition. The anatomical findings like actual defect size, visceral involvements and pulmonary hypoplasia are apparent only intra operatively while factors like cardiac lesions, birth weight and prematurity can be identified early. A fruitful outcome depends not only on successful repair but also in addressing each of the factors associated with prognosis. CDH has a well-known risk of congenital heart defects with poor prognosis.
